# Large-Vessel Vasculitis: Interobserver Agreement and Diagnostic Accuracy of ^18^F-FDG-PET/CT

**DOI:** 10.1155/2015/914692

**Published:** 2015-01-28

**Authors:** K. D. F. Lensen, E. F. I. Comans, A. E. Voskuyl, C. J. van der Laken, E. Brouwer, A. T. Zwijnenburg, L. M. Pereira Arias-Bouda, A. W. J. M. Glaudemans, R. H. J. A. Slart, Y. M. Smulders

**Affiliations:** ^1^Department of Internal Medicine and Institute for Cardiovascular Research (ICaR-VU), VU University Medical Center, 1007 MB Amsterdam, Netherlands; ^2^Department of Nuclear Medicine and Radiology, VU University Medical Center, 1007 MB Amsterdam, Netherlands; ^3^Department of Rheumatology, VU University Medical Center, 1007 MB Amsterdam, Netherlands; ^4^Department of Rheumatology, University Medical Center Groningen, 9700 RB Groningen, Netherlands; ^5^Department of Nuclear Medicine, Spaarne Hospital, 2130 AT Hoofddorp, Netherlands; ^6^Department of Nuclear Medicine, Rijnland Hospital, 2350 CC Leiderdorp, Netherlands; ^7^Department of Nuclear Medicine and Molecular Imaging, University Medical Center Groningen, 9700 RB Groningen, Netherlands

## Abstract

*Introduction*. ^18^F-FDG-PET visualises inflammation. Both atherosclerosis and giant cell arteritis cause vascular inflammation, but distinguishing the two may be difficult. The goal of this study was to assess interobserver agreement and diagnostic accuracy of ^18^F-FDG-PET for the detection of large artery involvement in giant cell arteritis (GCA). *Methods*. 31 ^18^F-FDG-PET/CT scans were selected from 2 databases. Four observers assessed vascular wall ^18^F-FDG uptake, initially without and subsequently with predefined observer criteria (i.e., vascular wall ^18^F-FDG uptake compared to liver or femoral artery ^18^F-FDG uptake). External validation was performed by two additional observers. Sensitivity and specificity of ^18^F-FDG-PET were determined by comparing scan results to a consensus diagnosis. *Results*. The highest interobserver agreement (kappa: 0.96 in initial study and 0.79 in external validation) was observed when vascular wall ^18^F-FDG uptake higher than liver uptake was used as a diagnostic criterion, although agreement was also good without predefined criteria (kappa: 0.68 and 0.85). Sensitivity and specificity were comparable for these methods. The criterion of vascular wall ^18^F-FDG uptake equal to liver ^18^F-FDG uptake had low specificity. *Conclusion*. Standardization of image assessment for vascular wall ^18^F-FDG uptake promotes observer agreement, enables comparative studies, and does not appear to result in loss of diagnostic accuracy compared to nonstandardized assessment.

## 1. Introduction

^18^F-Fluorodeoxyglucose Positron Emission Tomography (FDG-PET), usually combined with low-dose CT (FDG-PET/CT), may be used to detect vascular wall inflammation [1]. The most common causes of vascular wall inflammation are, by far, atherosclerosis and vasculitis, particularly giant cell arteritis (GCA). An infectious etiology (e.g., syphilis) is far less common [2–4]. Therefore, the primary goal for a clinician is to differentiate between GCA and atherosclerotic plaque inflammation when vascular wall ^18^F-FDG uptake is present [5]. GCA, a granulomatous vasculitis of unknown origin, may present clinically as temporal arteritis which is characterised by temporal headache, jaw claudication and visual symptoms. Large arteries are frequently involved [6]. However, temporal arteries are not always affected. This clinical phenotype, in which the clinical presentation is less specific, has been referred to as “silent,” “occult” or large-vessel vasculitis (LVV) [7, 8].

Several criteria for the qualitative (visual) assessment of FDG-PET for the detection of large artery involvement in GCA have been introduced, ranging from “increased” circumferential ^18^F-FDG uptake (i.e., not further specified) in a segment of the arterial wall to equal or more intense vascular wall uptake than liver uptake [9–12].

It has been recommended that only those with specific expertise and experience should assess vascular wall ^18^F-FDG uptake [9]. In clinical practice, and in our own experience, interobserver agreement among such experts is not always sufficient. However, this has been tested in only a few studies (Table 1) As low levels of agreement are problematic, since they preclude diagnostic accuracy [13], the primary objective of this study was to establish interobserver agreement for the visual assessment of vascular wall ^18^F-FDG uptake using various scoring methods. As a secondary objective, different FDG-PET/CT scoring methods were compared with the final clinical diagnosis.

## 2. Methods

### 2.1. Patient Characteristics

31 FDG-PET/CT scans were selected from the databases of the Department of Nuclear Medicine and PET Research of the VU University Medical Center (VUMC) and the Department of Nuclear Medicine and Molecular Imaging of the University Medical Center Groningen (UMCG). These FDG-PET/CT scans were performed in clinical practice in order to (1) determine the cause of inflammation of unknown origin (*n* = 12) (this was a subset of patients from a study addressing the value of FDG-PET in patients with systemic inflammation of unknown origin) [14], (2) investigate whether large-vessel vasculitis was present in patients with diagnosed temporal arteritis (*n* = 6) or polymyalgia rheumatic (PMR) (*n* = 7), or (3) do a follow-up procedure in patients with a history of cancer (*n* = 6) who were considered to be in clinical remission, from which a random selection was made.

The first two groups were selected as a high incidence of large-vessel vasculitis was expected. A diagnosis of temporal arteritis (according to the ACR criteria for GCA) [15] or PMR (in accordance with Healy criteria) was made by the treating physician. Two patients with a diagnosis of temporal arteritis and one patient with a diagnosis of PMR used prednisone prior to the FDG-PET/CT scan. The third (reference) group was selected because malignancy is the most common indication for an FDG-PET/CT scan making these scans easy to obtain. In addition, the incidence of large-vessel vasculitis was expected to be low.  Table 2 shows patient characteristics of these groups.

A Philips Gemini TOF (VUMC) and a Siemens Biograph (UMCG) PET/CT scanner (Philips Medical Systems, Eindhoven, Netherland and Siemens Medical Systems, Knoxville, TN) were used. A standardised protocol according to European Association of Nuclear Medicine (EANM) guidelines was used for the acquisition of scans [16]. In short, after fasting for at least four hours, whole-body (from head to knees) or total-body (from head to toes) PET-scans were acquired 60 (±5) minutes after intravenous injection of 3 MBq/kg ^18^F-FDG. A low-dose CT scan was acquired prior to the PET-emission scan for attenuation correction and anatomic localization. Each scan was given a specific study code before analysis assuring that observers were blinded for original scan report and clinical data. Finally, the observers were unaware of the number of patients with large-vessel vasculitis that were included. Blinding for medical center was impossible because PET/CT scans from two different vendors were used.

### 2.2. Image Analysis

Four observers used three distinct methods to visually (qualitatively) assess ^18^F-FDG vascular wall uptake (vascular uptake). These methods were applied in a consecutive manner in all scans and were consistently used by the observers. The level of experience varied among the observers, that is, 12, 8, 5 (three nuclear medicine physicians), and 2 years (one general physician working as a researcher in the field of PET/CT and large-vessel inflammation).

First, vascular uptake was assessed without using predefined criteria and was therefore based on first impression. This method was selected as it is often used in clinical practice, due to the absence of established observer criteria (qualitative and quantitative), such as comparing vascular uptake with uptake in other organs (e.g., the liver). Vascular wall uptake was scored as 1: normal, 2: atherosclerosis, or 3: large-vessel vasculitis (method (I)). The term large-vessel vasculitis was used as temporal artery involvement cannot be assessed using PET/CT due to a relatively low resolution of the scan and potential spill over from adjacent (physiological) brain ^18^F-FDG uptake [5].

Subsequently, the intensity of vascular uptake was systematically compared with the intensity of ^18^F-FDG liver uptake and scored as 0: absent, 1: less intense, 2: equally intense, or 3: more intense (method (II)).

The arterial segments that were studied in these first two methods included carotid, vertebral, subclavian, iliac, and femoral arteries, the aortic arch, and the ascending, descending, and abdominal aorta.

In the third method, femoral artery uptake was chosen as reference since the femoral artery is rarely involved in GCA and has a high incidence of atherosclerosis [17, 18]. The intensity of vascular uptake in the other arterial segments was compared with the intensity of femoral artery uptake and was scored as 0: less intense, 1: equally intense, or 2: more intense (method (III)).

The distribution pattern, either focal (in all segments <2 cm) or diffuse (at least one segment comprising more than 2 cm of contiguous vascular wall uptake), and presence of arterial calcification on low-dose CT were also scored.

After the first reading, a consensus meeting was held with the goal to clarify causes of disagreement.

Finally, interobserver agreement was calculated using definitions of large-vessel vasculitis for each of the applied methods. These definitions were

- first impression (no predefined criteria),
- diffuse vascular wall uptake,* equal to or higher than liver *uptake in more than one vascular segment,
- diffuse vascular wall uptake,* higher than liver* uptake in more than one vascular segment,
- diffuse uptake,* higher than femoral artery* uptake.

### 2.3. Clinical Diagnosis of Large Artery Involvement in GCA

To date, there is no clinical reference standard for a diagnosis of large artery involvement in GCA (either with or without temporal artery involvement). Furthermore, there is no consensus on which imaging modality or imaging criteria should be used. Therefore, in order to compare FDG-PET/CT results to a clinical diagnosis of large artery involvement in GCA, we defined the latter as

1. temporal arteritis according to ACR criteria [15], accompanied by an FDG-PET/CT scan that was unanimously classified as large-vessel vasculitis by all observers for at least one of the methods applied,
2. inflammation (i.e., elevated ESR) of unknown origin, not fulfilling ACR criteria for GCA, accompanied by FDG-PET/CT results that were unanimously classified as large-vessel vasculitis by all observers for at least two of the methods applied.

In both groups, a good clinical response to immunosuppressive therapy (prednisone), defined as rapid resolution of signs and symptoms, accompanied by normalisation of inflammatory parameters, was mandatory for the diagnosis. Additionally, no other diagnosis was allowed to have been established during a follow-up period of at least 3 months.

This clinical diagnosis was used to determine sensitivity and specificity for the 4 FDG-PET/CT scoring methods.

### 2.4. External Observers

Based on the results of the first four observers, the definitions of large-vessel vasculitis that showed high levels of agreement between observers and with the clinical diagnosis were used for external validation. The first four observers are employees at a university hospital and have specific interest and experience in large-vessel vasculitis and PET/CT reporting. Therefore, two community hospital nuclear medicine physicians were asked to score the same scans by these definitions. Again, interobserver agreement was determined.

### 2.5. Statistical Analysis

Levels of agreement were quantified using Fleiss' kappa (*κ*) for multiple raters and Cohen's kappa for two raters. *κ*-values are reported using the benchmarks of Landis and Koch (with 0.81–1 being almost perfect agreement; 0.61–0.8 being substantial agreement; 0.41–0.6 being moderate agreement; 0.21–0.4 being fair agreement; 0.01–0.2 being slight agreement; and ≤0 being poor agreement) [19]. The statistical program *R* was used to calculate Fleiss' kappa [20]. SPSS was used to calculate Cohen's kappa (SPSS version 20; SPSS inc.).

## 3. Results

### 3.1. First Impression Potentially Influences Comparison to Liver

After all scans were analysed, a remarkably high disagreement (i.e., 1/3 of all cases) was noticed when vascular uptake was compared to liver uptake. In a subgroup of patients vascular uptake was scored as less intense than liver uptake by some of the observers, whereas it was scored equally intense by other observers. During the consensus meeting, the observers concluded that in some cases vascular wall uptake was considered to be less intense than liver uptake based on their first impression that large-vessel vasculitis was not present. Therefore, a reanalysis of these scans was performed. Furthermore, the observers agreed that vascular uptake was only consistent with vasculitis if it showed a diffuse uptake pattern. The presence of calcification was ignored as it was present in all patients on low-dose CT scans.

### 3.2. Interobserver Agreement

**Figure 1:**
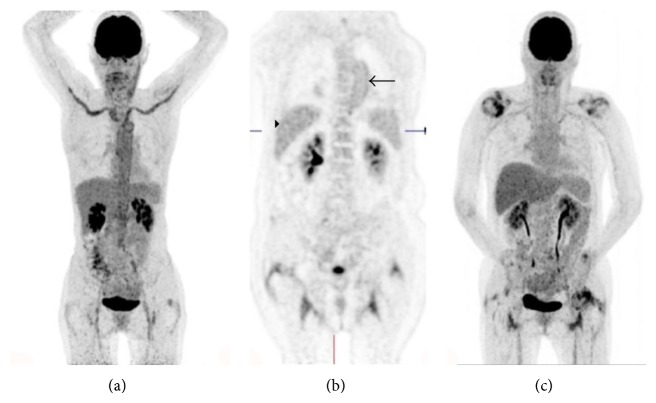
^18^F-FDG PET/CT scans showing: (a) Maximum intensity projection (MIP) image: scored as large-vessel vasculitis by all observers according to all methods, (b) coronal image: ^18^F-FDG uptake in descending aorta (arrow) scored as equal to liver uptake (arrowhead) by 2 observers and lower than liver uptake by 2 other observers, none of the observers scored higher than liver or femoral artery uptake, (c) MIP image of a PMR patient that was scored negative for large-vessel vasculitis by all observers. (Cerebral and urinary tract 18-^18^F-FDG uptake are physiological).

Table 3 shows the average number (and individual scores of all 4 observers) of scans that were scored as large-vessel vasculitis. A remarkably high number of large-vessel vasculitis diagnoses were made when definition (IIa) was used.  Figure 1 shows examples of FDG-PET/CT images in which there was complete agreement on absence or presence of large-vessel vasculitis according to all methods (Figures 1(a) and 1(c)) and an image in which there was disagreement (Figure 1(b)) according to method (II).

All observers agreed in 21 of 30 cases (70%) when assessing scans according to method (I). The corresponding Fleiss' kappa was 0,68 (0,72 without the results of the least experienced observer).

There was agreement between all observers in 19 of 30 patients (63%) when scoring according to method (IIa), and kappa was 0,78 (0,81 without results of the least experienced observer) (Table 3). When method (IIb) was applied, there was agreement between all observers in 29 of 30 cases (97%), resulting in a kappa of 0,96 (1 without the results of the least experienced observer).

Finally, for method (III), all observers agreed in 27 of 30 cases (90%) with a resulting kappa of 0,81 (1 without the results of the least experienced observer).

### 3.3. PET/CT Results and Clinical Diagnosis

In 4 patients (13%), we were unable to obtain sufficient follow-up data to ascertain a clinical diagnosis. One patient (from the group of inflammation of unknown origin who was suspected of large-vessel vasculitis after PET/CT) died the week after the scan was performed; autopsy was not performed. Three patients were lost to follow-up. In the remaining 27 patients, a clinical diagnosis of large-vessel vasculitis was established in 6 (22%) according to the previously mentioned criteria. The average sensitivity and specificity for all definitions are shown in Table 3. All definitions provide high sensitivity and specificity, with the exception of a low specificity for definition (IIa).

Of 6 patients with temporal arteritis, 4 had large artery involvement on FDG-PET/CT according to all observers. Two of these four patients had a negative temporal artery biopsy, whereas one biopsy was inconclusive (not arterial tissue). The two patients with a negative FDG-PET/CT scan, both, had a positive temporal artery biopsy. One of the 7 patients with PMR had large-vessel vasculitis on FDG-PET/CT when using definition (IIa), whereas no large-vessel vasculitis was present using definitions (IIb) and (III).

### 3.4. External Observers

Table 4 shows the results of the external observers. Definition (IIa) was not used in the external validation because of low specificity in the first analysis. Agreement between the 2 observers was high using definitions (I) and (IIb) and moderate using definition (III). Sensitivity of definition (I) was highest, whereas definitions (IIb) and (III) had the highest specificities.

## 4. Discussion

Our study suggests a preference for standardized observer criteria for the assessment of large-vessel vasculitis on ^18^F-FDG-PET/CT images. Among dedicated and experienced observers, assessment of diffuse vascular uptake that exceeds liver uptake provides the highest observer agreement. Additionally, sensitivity and specificity appear to be superior, although the results show considerable overlap in the confidence intervals, at least partly due to the limited number of patients in this study. Among less experienced observers, agreement was more or less similar for standardized criteria (i.e., comparing vascular uptake to liver uptake) versus “first impression.” Altogether, standardization of imaging criteria is equal to or superior to using first impression. In addition, standardization of imaging criteria will undoubtedly facilitate communication, both in clinical practice and in science, bearing in mind the increasing use of FDG-PET/CT in clinical practice and hence the assessment of vascular ^18^F-FDG uptake by observers with varying degrees of experience.

High interobserver agreement was suggested in two previous studies which also compared vascular uptake with liver uptake [10, 12]. In these observer-blinded studies, only two experienced observers assessed all scans, potentially reducing generalizability of their findings. In addition, only one method was used to assess uptake in these studies. As in most studies, vascular uptake was considered to reflect large-vessel vasculitis when uptake was equal to or higher than liver uptake. Our findings challenge the specificity of this criterion and suggest overestimation of the prevalence of large-vessel vasculitis using this definition. However, misclassification of our control patients cannot be excluded, since our criteria for the clinical diagnosis of large-vessel vasculitis were relatively strict. In addition, it has been suggested that immunosuppressive therapy may attenuate vascular uptake and hence require different interpretation (i.e., loss of sensitivity of definition (IIb)) [23]. Two of our patients (with a clinical diagnosis of temporal arteritis) used prednisone. In both, vascular uptake was not higher than liver uptake (one equal to and one less intense than liver uptake), and they did not fulfil our clinical criteria for large-vessel vasculitis. Regrettably, absence of histological proof, as is virtually always the case, precludes a definitive diagnosis. Finally, most of the earlier studies were performed using a stand-alone ^18^F-FDG-PET scanner. Modern (i.e., “hybrid”) PET/CT scans may be more sensitive, due to attenuation correction, thereby potentially detecting vascular uptake (either resulting from physiological processes, atherosclerosis, or low-grade/subclinical inflammation/vasculitis) in large arteries in “healthy” controls [34].

Our study has some strengths and limitations. One of the strengths is that observer agreement was studied in a group of observers with a varying degree of experience, enhancing the generalizability of the results. Additionally, external validation by nonacademical nuclear medicine physicians confirmed the results, which is important as many studies addressing this topic are performed solely in an academic setting. In clinical practice, FDG-PET/CT scans will be assessed by nuclear medicine physicians or radiologists with a varying amount of clinical experience. The clinical reference standard that we constructed is also a strength of this study. Although we do realise that the definitions we used to establish a clinical diagnosis of large-vessel vasculitis are not universally accepted diagnostic criteria, we at least made a serious attempt to relate imaging tests to a clinical diagnosis. Our proposed criteria incorporate the most important characteristics of GCA (clinical signs and symptoms, inflammation and rapid response to steroids) and a consensus among multiple observers regarding imaging characteristics of vasculitis in large arteries. This approach enabled us to establish that changing the cut-off value for ^18^F-FDG uptake (higher than liver uptake as opposed to equal to or higher) did not seem to affect sensitivity of the clinical diagnosis but increased specificity. Another strength is the application of different criteria in a single study to investigate whether one definition possesses both high interobserver agreement and diagnostic accuracy. In the current study, vascular wall uptake was also compared with femoral artery uptake, as we have experienced that, in the elderly (i.e., patients over 50 years of age), the femoral artery invariably displays ^18^F-FDG uptake. It remains to be elucidated whether this results from atherosclerotic plaque inflammation, which is known to be common in the elderly [18], or from physiological activity. Large-vessel vasculitis infrequently involves femoral arteries [17]. Although using vascular wall uptake in the femoral artery as reference has a theoretical drawback of a lower sensitivity (when the femoral artery is involved in the disease process, as observed in our study), its specificity and degree of observer agreement render it a good alternative when liver uptake is unclear (e.g., in patients with liver diseases causing nondiffuse liver uptake).

A limitation is the absence of a true reference standard for the diagnosis of large-vessel vasculitis, which would need to be histopathological evidence. Obviously, our clinical diagnosis comprises the test under study which may introduce bias. However, as a “practical gold standard” to establish large-vessel vasculitis is currently not present, we believe that this approach may be a first step towards establishing such a gold standard. We are inclined to think that the criteria we used for a patient to be classified as a large-vessel vasculitis patient were appropriate.

The limited sample size may also be considered a limitation. Although we included patients with inflammation of unknown origin and apparently healthy patients (after follow-up for malignancy) as controls, which further enhances the external validity of our study, the limited size of all groups warrants corroboration in a larger group of similar patients. Finally, it has been suggested that not two but four characteristics might differentiate vasculitis from atherosclerotic plaque inflammation [5]. These include intensity of vascular wall uptake (vasculitis being more intense), distribution pattern (vasculitis being more diffuse and affecting primarily the thoracic arteries, subclavian, and vertebral arteries, whereas atherosclerotic plaque inflammation is supposed to be focal, mainly affecting the abdominal aorta and the iliofemoral arteries), calcification (atherosclerosis patients having more calcification), and quantification of FDG uptake. Our study mainly addressed the first two characteristics. Calcification was also assessed in all patients. However, as 28 of 30 patients displayed calcification on low-dose CT (results not shown) this characteristic was not expected to contribute to differentiation. Moreover, quantitative assessment in patients with atherosclerotic disease has suggested that vascular inflammation is lower in vascular segments showing calcification [35–37], although studies are not unequivocal [38]. Using a semiquantitative approach may help to incorporate calcification in the differentiation between patients with GCA and atherosclerosis [38]. Standardized uptake values (SUVs) may be calculated in regions of interest (ROIs) in the vascular wall (whether SUVmax or SUVmean should be used remains to be established) and in the liver to determine ratios.

## 5. Conclusion

In conclusion, predefined standardized criteria (comparing vascular uptake to liver uptake) have high interobserver agreement and probably have good diagnostic accuracy for large-vessel vasculitis. All patients with a clinical diagnosis of large-vessel vasculitis displayed diffuse vascular wall uptake higher than liver uptake. We recommend that observers consider scans with these characteristics to be consistent with large-vessel vasculitis when using modern PET/CT scanners. In case of irregular liver uptake, femoral artery uptake may be used as an alternative reference standard, bearing in mind that sensitivity might be slightly lower if the femoral artery is involved in the disease process. Finally, these results may not apply to patients that used steroids prior to the FDG-PET/CT scan. Future studies need to address the effect of steroids on vascular uptake (i.e., establishing time interval between start of steroids and resolution of characteristics on FDG-PET/CT) and the potential value of semiquantitative methods (SUVs).

## Figures and Tables

**Table 1:** Overview of articles reporting imaging findings in GCA/large-vessel vasculitis and assessment of observer agreement of visual assessment. (n.r.: not reported, ^18^F-FDG-PET: ^18^F-fluorodeoxyglucose positron emission tomography, MRI: magnetic resonance imaging, CT: computed tomography, and US: ultrasound).

Article (year of publication)	Imaging modality	Number of observers	Observer agreement
Blockmans et al., 2000 [21]	^ 18^F-FDG-PET	4 (2 teams)	n.r.
Blockmans et al., 2006 [22]		2	n.r.
Walter et al., 2005 [12]		2	93% (28/30)

Henes et al., 2008 [9]	^18^F-FDG-PET/CT	At least 2	n.r.
Lehmann et al., 2011 [10]		2	85% (Cohen's kappa 0.7)
Papathanasiou et al., 2012 [23]		2	n.r.
Fuchs et al., 2012 [3]		3	n.r.

Brodmann et al., 2004 [24]	^ 18^F-FDG-PET and US	1	n.r.

Meller et al., 2003 [11]	^ 18^F-FDG-PET and MRI	2	n.r.
Scheel et al., 2004 [25]		2	n.r.
Both et al., 2008 [26]		2	n.r.

Agard et al., 2008 [27]	CT	1	n.r.
Marie et al., 2009 [28]		1	n.r.
Prieto-González et al., 2012 [6]		2	98%

Schmidt et al., 2002 [29]	US	2	89%
Schmidt et al., 2008 [30]		?	n.r.
DE et al., 2009 [31]		29	0.847 (kappa)

Narvaez et al., 2005 [32] Koenigkam-Santos et al., 2011 [33]	MRI	n.r. 2	n.r. 0.73 (kappa)

**Table 2:** Patient characteristics, total and ordered by group.

	Total (*n* = 31)	Inflammation of unknown origin (*n* = 12)	Temporal arteritis (*n* = 6)	Polymyalgia rheumatica (*n* = 7)	Control group (*n* = 6)
Age (years)^*^	70 (12)	73 (13)	67 (10)	73 (7)	62 (13)
Sex (female)^•^	65%	58%	100%	57%	50%
ESR (mm/h)^*^	70 (32)	79 (27)	64 (42)	58 (30)	Unknown
BMI (kg/m^2^)^*^	23,4 (7,3)	23,7 (8,6)	23,3 (4,6)	21,6 (9,9)	25 (1,7)

^*^Mean (standard deviation), ^•^percentage.

**Table 3:** Average number of vasculitis PET/CT scores (individual observer scores), Fleiss' kappa, sensitivity, and specificity (95%-CI) according to the different methods applied.

Method	Average number of vasculitides (*x*_1_, *x*_2_, *x*_3_, *x*_4_)	Fleiss' kappa	Sensitivity^*^	Specificity^*^
(I) First impression	9 (6, 9, 10, 11)	0,68	92% (52–98%)	90% (70–97%)

(IIa) Diffuse uptake, ≥liver uptake	16 (14, 16, 17, 18)	0,78	100% (61–100%)	60% (39–78%)

(IIb) Diffuse uptake, >liver uptake	7 (7, 7, 7, 8)	0,96	100% (61–100%)	98% (82–100%)

(III) Diffuse uptake, >femoral artery uptake	7 (6, 6, 6, 10)	0,81	80% (41–94%)	96% (79–99%)

^*^The average sensitivity and specificity of the results of the 4 observers (*x*_1_–*x*_4_) were calculated.

**Table 4:** Average number of vasculitis PET/CT scores (individual observer scores), Cohen's kappa, sensitivity, and specificity (95%-CI) according to the different methods applied.

Method	Average vasculitis score (*x*_1_, *x*_2_)	Cohen's kappa	Sensitivity^*^	Specificity^*^
(I) First impression	10 (9, 11)	0,85	100% (61–100%)	88% (67–96%)

(IIb) Diffuse uptake, >liver uptake	6 (5, 7)	0,79	83% (46–95%)	100% (84–100%)

(III) Diffuse uptake, >femoral artery uptake	7 (7, 7)	0,63	83% (44–97%)	93% (73–98%)

^*^The average sensitivity and specificity of the results of the 2 observers (*x*_1_–*x*_2_) were calculated.
